# Taking Age-Friendly Campus Efforts to New PEAKS

**DOI:** 10.1093/geroni/igab046.1495

**Published:** 2021-12-17

**Authors:** Pamela Elfenbein

**Affiliations:** University of North Georgia, Gainesville, Georgia, United States

## Abstract

The University of North Georgia’s Personal Enrichment, Action, Knowledge Series (PEAKS) was developed as a monthly series of engaging and seasonally appropriate presentations and activities designed to expressly meet the needs of the region’s large and quickly growing older adult population during the Covid-19 pandemic and resultant isolation. The PEAKS programs are available virtually to allow for broad participation throughout the 30 county UNG service region. While we developed PEAKS to specifically reach-out to older adults in our region, we have found that our audience is actually multi-generational, actively engaging with our speakers and one another. AFU underpinnings for PEAKS include Principle #8 - To enhance access for older adults to the university's range of health and wellness programs and its arts and cultural activities; and #9 - To engage actively with the university's own retired community.

